# Associations between cardiorespiratory fitness and weight loss in patients with severe obesity undergoing an intensive lifestyle intervention program: retrospective cohort study

**DOI:** 10.1186/s12902-019-0394-z

**Published:** 2019-07-01

**Authors:** Jarle Berge, Øyvind Støren, Jens K. Hertel, Espen Gjevestad, Milada Cvancarova Småstuen, Jøran Hjelmesæth

**Affiliations:** 1 Morbid Obesity Centre, Vestfold Hospital Trust, Box 2168, 3103 Tønsberg, Norway; 2 Clinic Medicine and Rehabilitation, Vestfold Hospital Trust, Tønsberg, Norway; 3 Nature, health and environment, University of Southeast, Bø in Telemark, Norway; 4 Norwegian Police University College, Stavern, Norway; 5 Department of Endocrinology, Morbid Obesity and Preventive Medicine, Institute of Clinical Medicine, University of Oslo, Oslo, Norway

**Keywords:** VO_2max_, Weight change, Severe obesity, Lifestyle intervention

## Abstract

**Background:**

To assess the association between cardiorespiratory fitness (CRF) and weight changes in treatment seeking patients with severe obesity who underwent a 1-year intensive lifestyle intervention (ILI) program.

**Methods:**

Retrospective cohort study conducted at a tertiary care outpatient rehabilitation center from November 1, 2013 through January 1, 2017. CRF was measured as maximal oxygen consumption during a maximal oxygen uptake (VO_2max_) test on a treadmill or bicycle at baseline and after 3 months.

**Results:**

A total of 180 patients had a baseline mean (SD) BMI 41.1 (4.8) kg/m^2^ and CRF of 79.4 (14.9) mL·kg^-0.75^·min^− 1^. Patients with a baseline CRF above median achieved a greater 3-month and 1-year weight loss compared with patients with CRF below median; mean (95% CI) 2.5 kg (1.3, 3.8) and 4.0 kg (0.8, 7.2), respectively. In addition, patients with 3-month changes of CRF above median had 4.0 kg (0.9, 7.1) greater weight loss at 1-year follow-up than those below median.

**Conclusions:**

Among patients with severe obesity who underwent a 1-year ILI program, higher baseline CRF was associated with significantly larger weight loss after 3 months and 1 year. In addition, those with higher initial 3-month CRF changes had greater weight loss at 1 year.

**Trial registration:**

Retrospectively registered in Regional Committees for Medical and Health Research Ethics (REC) south east September 22, 2016 (2016/1414) and clinicaltials.gov August 13, 2018 (identifier: NCT03593798).

**Electronic supplementary material:**

The online version of this article (10.1186/s12902-019-0394-z) contains supplementary material, which is available to authorized users.

## Background

Low cardiorespiratory fitness (CRF) is associated with lower energy expenditure [1, 2], higher body mass index (BMI) [3, 4] and increased waist circumference [5]. Patients with severe obesity therefore often have reduced CRF [3, 4, 6, 7]. Since lower CRF can result both in a lower daily activity level [8] and a lower potential for energy expenditure during activities [9], improving CRF could potentially induce weight reduction [10]. In line with this, improvements in CRF have been associated with decreased amounts of subcutaneous fat, visceral fat, liver fat and total fat mass, as well as decreased waist and hip circumference [11–14]. Importantly, exercise and increased CRF seem to both promote greater fat mass loss and assist preservation of lean mass, compared with energy restriction alone during weight loss interventions [15]. Further, a large prospective cohort study showed that improvements in CRF were associated with attenuated age-related weight gain in healthy middle-age adults [16].

The most used measure of CRF is maximal oxygen consumption (VO_2max_). Each liter of oxygen consumed liberates approximately 5 kcal, dependent upon the intensity of work [9]. This means that at any given relative work intensity, a higher VO_2max_ is accompanied with a higher energy expenditure. Energy expenditure has been shown to correlate with the rate of body weight change [17], and, accordingly, CRF may affect body weight change. However, to our knowledge, no previous study has addressed the association between CRF and body weight change in patients with severe obesity undergoing an intensive lifestyle intervention (ILI) program. Thus, the potentially predictive effect of CRF on weight loss is uncertain.

The primary aim of the present study was to investigate the association between CRF and weight loss in treatment seeking patients with severe obesity, who participated in a 1-year ILI program at a tertiary care center. We hypothesized that CRF above median at baseline would be associated with greater weight loss at the 3-month and 1-year follow-up, since a higher capacity for oxygen consumption will imply a higher capacity for energy expenditure, and that increasing CRF the first 3 months would be associated with greater level of weight loss at the 1-year follow-up.

## Methods

### Study design

This is a retrospective analysis of data from a cohort of patients with severe obesity who underwent a 1-year ILI program at a tertiary care outpatient rehabilitation center in Norway between November 1, 2013 and January 1, 2017.

### Participants

A total of 195 consecutive patients with severe obesity (BMI ≥40 kg/m^2^, or 35–39.9 kg/m^2^ with at least one obesity related co-morbidity) seeking treatment between 2013 and 2015 at the Clinic of Physical Medicine and Rehabilitation, Vestfold Hospital Trust, were eligible for this study. These patients were informed through an opt-out consent letter which stated that previously collected information during the ILI program would be used to answer the research questions stated in this project. Patients had a minimum of three weeks to opt-out of the study, and only two patients reserved usage of their personal information in this study.

Patient data from baseline to approximately 3-month and 1-year follow-up were included in the analyses. The study was approved by the Regional Committees for Medical and Health Research Ethics (REC) South East (2016/1414) and registered at clinicaltials.gov (identifier: NCT03593798).

### Interventions

The ILI program has been described in detail previously (Additional file 1) [18]. In brief, the program included both calorie restriction and physical exercise, and it aimed to help patients change their lifestyle, improve their CRF, and to achieve at least 5–10% weight loss. The patients received a dietary plan with an energy restriction of approximately 1000 kcal per day and were informed to follow restriction throughout the year. They had regular exercise sessions to increased energy expenditure during 3-month and were informed to continue regular exercise sessions throughout the year. The 3-month ILI program consisted of group- and individual treatments 3 days a week, including two supervised exercise sessions (duration 60–90 min.) per day and two lectures on healthy lifestyle behavior per day (Time schedule- Additional file 1). Exercise sessions included one weight-bearing activity and one water- based exercise per day. The exercise sessions were mainly of moderate to high aerobic intensity (4–8 metabolic equivalents), but also included some resistance sessions (75–90% of one repetition maximum) [19]. From month 3 to 12, patients attended monthly sessions including group exercise, group lectures and individual sessions with an interdisciplinary team that included a nurse, a medical doctor and physical educators.

### Outcome and predictor variables

The primary outcomes were changes in body weight between baseline and the 3-month and 1-year follow-up. Changes in waist circumference were also assessed. Baseline CRF and 3-month change in CRF were treated as exposure variables and possible predictors of weight loss.

### Measurements

CRF measured as VO_2max_ [20] was assessed at baseline and after 3 months. VO_2max_ is expressed as the absolute volume of oxygen consumed in liters (L/min) or related to body weight (mL·kg^− 1^·min^− 1^) [20]. VO_2max_ expressed relative to body weight (mL·kg^− 1^·min^− 1^) has been shown to underestimate CRF in heavy subjects, and overestimate CRF in light subjects. VO_2max_ scaled relative to the body weight raised to the power of 0.75 (mL·kg^-0.75^·min^− 1^) has eliminated the differences [21–24]. In order to account for VO_2max_ related to body size and weight, the results of VO_2max_ were allometrically scaled to mL·kg^-0.75^·min^− 1^.

The VO_2max_ test was mainly performed as an incremental treadmill test on Woodway ELG 55 (Waukesha, Germany) or, for patients with walking restrictions (*n* = 8), as an incremental bicycle test (Lode Corival V3, Lode BV, Groningen, Netherlands). For the treadmill test, velocity (0.5 km·h^− 1^) or inclination (1%) were increased every 30 s, until voluntary exhaustion. During the bicycle test brake power was increased by 25 W every 30 s until voluntary exhaustion. The duration of the test ranged between 4 and 10 min. Voluntary exhaustion, respiratory exchange ratio ≥ 1.05, calculated heart rate ≥ 95% of maximal heart rate, Borg scale ≥17 or flattening of VO_2_ curve were used to evaluate if VO_2max_ had been achieved. VO_2max_ was set as the highest sum of three consecutive 10 s measurements. Maximal heart rate was set as the highest observed value. Oxygen uptake was registered using the Jaeger oxycon pro ergospirometry test system (Jaeger Oxycon Pro JLAB 5.x, Hoechberg, Germany). The system includes a mixing chamber with oxygen (O_2_) and carbon dioxide (CO_2_) analyzed continuously every 10 s. Before each test, the device was checked and calibrated with room air and a certified gas containing 16% O_2_ and 4% CO_2_. Volume calibration was performed before each test, with an automatic syringe (2-L/s high flow and 0.2 L/s low flow) in Jaeger oxycon pro ergospirometry test system. A face mask (Hans Rudolph V2 mask) with different size (petit, XS, S, M) was used to collect expired air during the test. HR was collected continuously through the tests with Polar WearLink+ H7 bluetooth and Polar RCX 5 (Polar RCX 5, Polar Electro OY, Finland). The patients were instructed to refrain from eating 2 h and smoking 4 h before measurements, as well as to only drink water and to refrain from high intensity exercise in the last 24 h prior to measuring.

Body weight was measured with patients wearing light clothing and no shoes on Scanvaegt DS-530 (Århus, Denmark). Height was measured using a Seca (B.D.G.M) wall-mounted measuring tape. BMI was calculated as weight in kilograms divided by height in meters squared. Waist circumference was measured midway between the bottom edge of the lower rib and upper iliac crest in the horizontal plane. All anthropometric measures were performed at baseline, and after 3 months and 1 year.

### Sample size

No formal sample-size calculations were performed. All patients who attended the outpatient rehabilitation center during the study period of November 1, 2013 to January 1, 2017, in addition to those who completed follow-up at baseline and 3-month, baseline and 1-year, or at baseline, 3-month and 1-year follow-up, were asked to participate.

### Statistical analyses

All statistical analyses were performed using the Statistical Package for Social Sciences (SPSS) version 23 (Chicago, IL). Baseline CRF values were tested for normal distribution by visual inspection of QQ-plot, and were found to be normally distributed. Descriptive statistics are presented as mean and standard deviation (SD) unless otherwise specified. Independent samples t-tests or Fisher’s exact test as appropriate were used to analyze differences in outcomes between groups. Analysis of covariance (ANCOVA) adjusted for age, gender and baseline value were performed in order to identify potential differences between groups from baseline to 3-month and 1-year follow-up. Pearson bivariate correlation tests were used in order to investigate the correlations between initial CRF and weight reduction, and between improvement in CRF and weight reduction. In order to evaluate the strength of the correlations, Cohen’s f^2^ tests were performed. All P- values < 0.05 were considered statistically significant and all tests were two-sided.

## Results

**Fig. 1:**
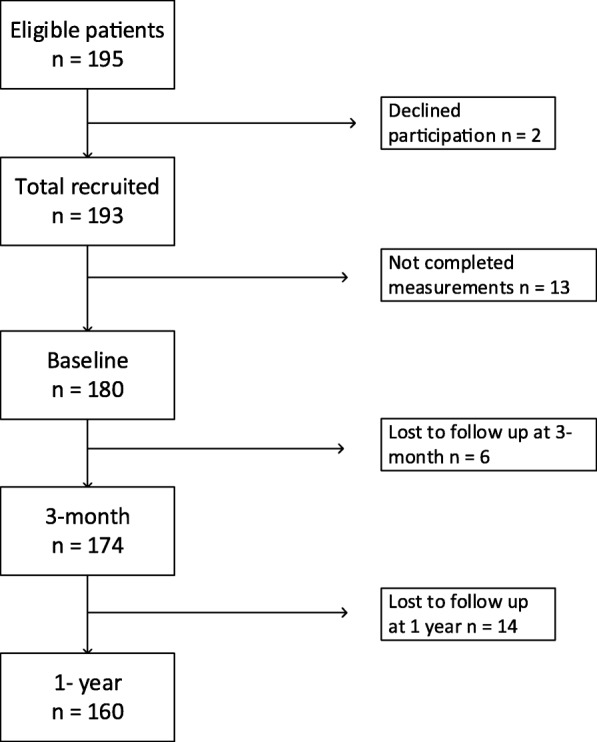
Flow chart

Of 195 patients eligible for this study, two patients declined participation, and 13 patients did not complete measurements at baseline (Fig. 1). The remaining 180 patients underwent baseline measurements, six of whom did not complete the first 3 months (median 11 weeks, range 9–16) of the ILI program, leaving 174 patients to be included in the analysis of the 3-month anthropometric data. Further, 14 patients did not complete measurements at 12 months (median 11 months, range 9–14) leaving 160 patients to be included in the analysis of the 1-year anthropometric data (Fig. 1). In addition, 143 patients completed CRF measurements at baseline and the 3-months follow-up, and weight measurements at the 1-year follow-up.

Overall, the 180 (79% female) patients had a baseline mean (SD) age of 43.5 (9.4) years, BMI 41.1 (4.8) kg/m^2^, body weight 118.8 (19.2) kg and CRF 79.4 (14.9) mL·kg^-0.75^·min^− 1^ (Table 1). Males had significantly higher age, body weight, waist circumference and CRF than females (Table 1).

**Table 1:** Baseline demographics, anthropometrics and cardiorespiratory fitness among treatment seeking patients according to gender

Variable	Total (*n* = 180)	Males (*n* = 38)	Females(*n* = 142)	*P*-value
Caucasian	174 (97%)	37 (97%)	137 (96%)	
Age (years)	43.5 (9.4)	47.0 (10.2)	42.6 (9.0)	0.010
BMI (kg/m^2^)	41.1 (4.8)	42.3 (5.0)	40.8 (4.7)	0.086
Weight (kg)	118.8 (19.2)	137.3 (20.5)	113.8 (15.6)	< 0.001
Waist circumference (cm)	116.8 (12.9)	130.0 (12.1)	113.4 (10.7)	< 0.001
VO_2max_ (mL·kg^-0.75^·min^− 1^)	79.4 (14.9)	86.5 (18.9)	77.4 (13.1)	0.007
VO_2max_ (mL·kg^−1^·min^− 1^)	24.2 (4.7)	25.4 (5.8)	23.9 (4.3)	0.072
VO_2max_ (L·min^−1^)	2.85 (0.63)	3.45 (0.78)	2.68 (0.46)	< 0.001

Data are presented as numbers (%) or mean (SD), independent samples t-test

**Fig. 2:**
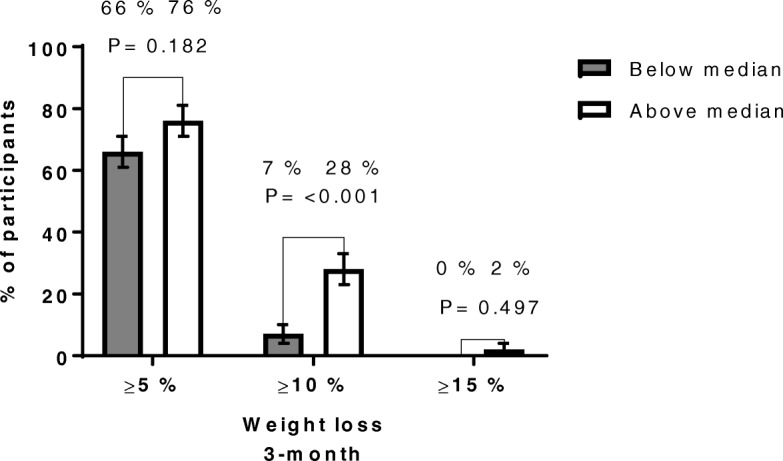
Proportions of patients in the two groups who at 3-month follow-up achieved at least 5, 10% or 15% weight loss. Data are presented as percentage of participants with standard error of the mean (SEM), either above or below baseline CRF median, who achieved at least 5, 10% or 15% weight loss at 3-month follow-up. Baseline CRF above median, *N* = 87. Baseline CRF below median, N = 87. In the above median CRF group, the number of patients who achieved at least 5, 10% or 15% was 66 (76%, SEM 5%), 24 (28%, SEM 5%), 2 (2%, SEM 2%), respectively. In the below median CRF group, the number of patients who achieved at least 5, 10% or 15% was 57 (66%, SEM 5%), 6 (7%, SEM 3%), 0 (0%, SEM 0%), respectively

Compared with patients who had a baseline CRF below median (78.3 mL·kg^-0.75^·min^− 1^), those with a CRF above median had a mean (95% CI) 2.5 (1.3–3.8) kg greater 3-month weight loss (Table 2). Further, the proportion (95% CI) of patients achieving a 3-month weight loss of ≥10% was significantly higher in those with a baseline CRF above median compared with those below median 28% (18–37) vs 7% [2–12] (Fig. 2). In addition, higher baseline CRF was weakly correlated with 3-month weight loss, r = − 0.17, *P* = 0.026 (Cohen’s f^2^ = 0.03) (Additional file 2: Figure S1).

**Table 2:** Anthropometrics, cardiorespiratory fitness and weight changes at the 3-mo follow-up

Cardiorespiratory fitness (mL·kg^-0.75^·min^− 1^)					
	Below median (*N* = 87)	Above median (N = 87)	*P*- value	Adjusted differences between groups	*P*- value adjusted differences between groups
Baseline					
Age (year)	45.8 (10.1)	41.2 (7.9)	0.001		
Gender (female)	75 (86.2%)	63 (72.4%)	0.039		
Weight (kg)	119.9 (18.9)	117.4 (19.6)	0.401		
BMI (kg/m^2^)	42.2 (4.5)	40.0 (5.0)	0.003		
Waist circumference (cm)	119.7 (11.7)	113.8 (13.2)	0.004		
VO_2max_ (mL·kg^-0.75^·min^−1^)	68.0 (8.9)	91.1(10.0)	< 0.001		
VO_2max_ (mL·kg^− 1^·min^− 1^)	20.7 (2.9)	27.8 (3.4)	< 0.001		
VO_2max_ (L·min^−1^)	2.46 (0.44)	3.24 (0.53)	< 0.001		
3-mo					
Weight (kg)	112.6 (18.0)	108.5 (18.4)	0.134		
BMI (kg/m^2^)	39.6 (4.3)	37.0 (4.9)	< 0.001		
Waist circumference (cm)	111.2 (11.9)	104.7 (12.8)	0.001		
VO_2max_ (mL·kg^-0.75^·min^−1^)^#^	77.9 (10.8)	101.5 (15.0)	< 0.001		
VO_2max_ (mL·kg^−1^·min^− 1^)^#^	24.1 (3.4)	31.5 (4.9)	< 0.001		
VO_2max_ (L·min^−1^)^#^	2.68 (0.47)	3.40 (0.67)	< 0.001		
Changes 3-mo					
Weight change (%)	−6.0 (−6.7, −5.4)	−7.6 (−8.3, −6.9)	0.002		
Weight change (kg)	−7.3 (− 8.1, − 6.4)	−9.0 (−9.9, − 8.0)	0.008	2.5 (1.3, 3.8)	< 0.001
BMI change (kg/m^2^)	−2.6 (−2.8, −2.3)	−3.0 (−3.3, − 2.7)	0.026	0.9 (0.4, 1.3)	< 0.001

Data are presented as mean (SD or CI 95%) or numbers (%). Groups are divided by VO_2max_ median (78.3 mL·kg^-0.75^·min^− 1^). Differences between groups analyzed using independent samples t-test or Fisher’s exact test (gender) as appropriate. Adjusted differences between groups in term of changes were analyzed using analysis of covariance (ANCOVA) including age, gender and baseline value of outcome variable as covariates. ^#^, *N* = 74 below median, *N* = 82 above median

**Fig. 3:**
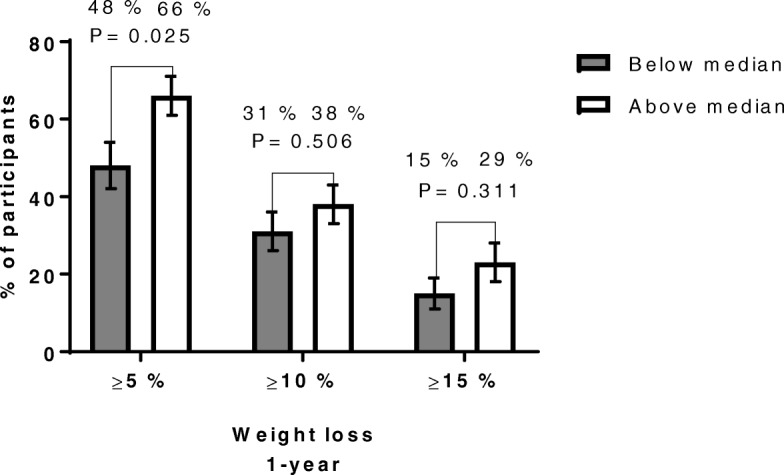
Proportions of patients in the two groups who at 1-year follow-up achieved at least 5, 10% or 15% weight loss. Data are presented as percentage of participants with standard error of the mean (SEM), either above or below baseline CRF median, who achieved at least 5, 10% or 15% weight loss at 1-year follow-up. Baseline CRF above median, *N* = 80. Baseline CRF below median, N = 80. In the above median CRF group, the number of patients who achieved at least 5, 10% or 15% was 53 (66%, SEM 5%), 30 (38%, SEM 5%), 18 (23%, SEM 5%), respectively. In the below median CRF group, the number of patients who achieved at least 5, 10% or 15% was 38 (48%, SEM 6%), 25 (31%, SEM 5%), 12 (15%, SEM 4%), respectively

Patients with baseline CRF values above median had a mean of 4.0 (0.8, 7.2) kg greater 1-year weight loss than those with CRF values below median (Table 3). However, the proportion of patients achieving a 1-year weight loss of ≥10% was not significantly higher in those with a baseline CRF above median compared with those below median 38% (27–48) vs 31% (21–42) (Fig. 3). In addition, higher baseline CRF was weakly correlated with 1-year weight loss, r = − 0.17, *P* = 0.028 (Cohen’s f^2^ = 0.03) (Additional file 2: Figure S2).

**Table 3:** Anthropometrics, cardiorespiratory fitness and weight changes at the 1-yr follow-up

Cardiorespiratory fitness (mL·kg^-0.75^·min^− 1^)					
	Below median (*n* = 80)	Above median (n = 80)	*P*- value	Adjusted differences between groups	*P*- value adjusted differences between groups
Baseline					
Gender (female)	68 (85.0%)	55 (68.7%)	0.024		
Age (years)	45.8 (10.1)	41.9 (7.5)	0.006		
Weight (kg)	120.2 (19.5)	117.7 (19.6)	0.427		
BMI (kg/m^2^)	42.2 (4.6)	39.9 (4.8)	0.002		
Waist circumference (cm)	119.8 (12.2)	114.7 (13.8)	0.015		
VO_2max_ (mL·kg^-0.75^·min^−1^)	67.8 (9.1)	91.5 (10.2)	< 0.001		
VO_2max_ (mL·kg^−1^·min^− 1^)	20.6 (2.9)	27.9 (3.4)	< 0.001		
VO_2max_ (L·min^−1^)	2.46 (0.45)	3.26 (0.54)	< 0.001		
1-yr					
Weight (kg)	112.5 (20.8)	107.4 (21.3)	0.127		
BMI (kg/m^2^)	39.5 (5.4)	36.4 (5.8)	< 0.001		
Waist circumference (cm)	109.3 (16.4)	103.4 (16.2)	0.022		
Changes 1-yr					
Weight change (%)	−6.4 (−8.1, −4.8)	−8.9 (−10.8, − 7.0)	0.050		
Weight change (kg)	−7.7 (− 9.6, − 5.7)	−10.3 (− 12.5, − 8.1)	0.078	4.0 (0.8, 7.2)	0.014
BMI change (kg/m^2^)	−2.7 (− 3.4, − 2.0)	− 3.5 (− 4.2, − 2.7)	0.118	1.3 (0.1, 2.4)	0.029

Data are presented as mean (SD or CI 95%) or numbers (%). Groups are divided by VO_2max_ median (78.3 mL·kg^-0.75^·min^− 1^). Differences between groups analyzed using independent samples t-test or Fisher’s exact test (gender) as appropriate. Adjusted differences between groups in term of changes were analyzed using analysis of covariance (ANCOVA) including age, gender and baseline value of outcome variable as covariates

**Fig. 4:**
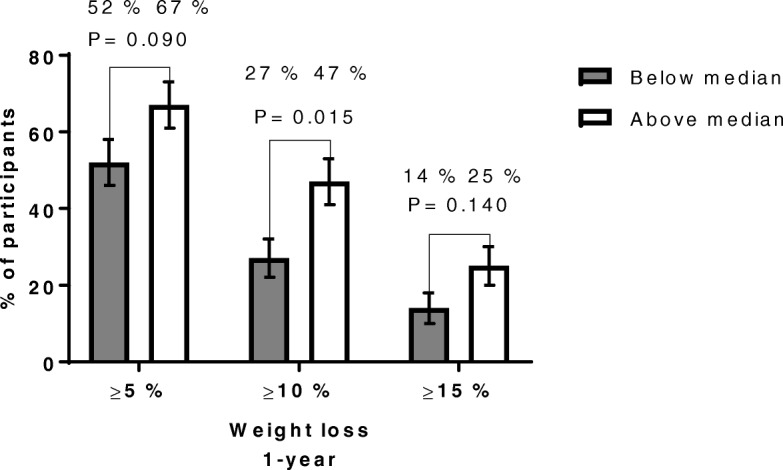
Proportions of patients in the two groups, who achieved median 3-month percentage increases in CRF, who at 1-year follow-up achieved at least 5, 10% or 15% weight loss. Data are presented as percentage of participants with standard error of the mean (SEM), either above or below median 3-month percentage increases in CRF, who achieved at least 5, 10% or 15% weight loss at 1-year follow-up. 3-month percentage increases in CRF above median, *N* = 72. 3-month percentage increases in CRF below median, *N* = 71. In the above median CRF group, the number of patients who achieved at least 5, 10% or 15% was 48 (67%, SEM 6%), 34 (47%, SEM 6%), 18 (25, 5%), respectively. In the below median CRF group, the number of patients who achieved at least 5, 10% or 15% was 37 (52%, SEM 6%), 19 (27%, SEM 5%), 10 (14%, SEM 4%), respectively

Patients who achieved an above median 3-month increase in CRF had 4.0 (0.9–7.1) kg greater weight loss at 1-year follow-up than those below median (Table 4). Further, the proportion of patients achieving a 1-year weight loss of ≥10% was significantly higher in those with CRF changes above median compared with those below median 47% (35–59) vs 27% (16–37) (Fig. 4). In addition, the 3-month change in CRF was weakly correlated with 1-year weight loss, r = − 0.26, *P* = 0.002 (Cohen’s f^2^ = 0.07) (Additional file 2: Figure S3).

**Table 4:** Anthropometrics, cardiorespiratory fitness and weight changes at the 1-yr follow-up in patients percentage increases 3-mo cardiorespiratory fitness

Percentage increased cardiorespiratory fitness, (mL·kg^-0.75^·min^− 1^)					
	Below median (*n* = 71)	Above median (*n* = 72)	*P*- value	Adjusted differences between groups	*P*- value adjusted differences between groups
Baseline					
Gender (female)	58 (81.7%)	54 (75.0%)	0.418		
Age (years)	44.3 (8.7)	42.3 (8.6)	0.156		
Weight (kg)	116.9 (18.6)	120.2 (19.3)	0.303		
BMI (kg/m^2^)	40.7 (4.7)	41.2 (4.7)	0.548		
Waist circumference (cm)	116.0 (12.6)	117.2 (13.0)	0.576		
VO_2max_ (mL·kg^-0.75^·min^−1^)	81.7 (13.2)	79.6 (15.6)	0.374		
VO_2max_ (mL·kg^−1^·min^− 1^)	25.0 (4.4)	24.2 (4.8)	0.307		
VO_2max_ (L·min^−1^)	2.89 (0.54)	2.88 (0.68)	0.919		
3-mo					
Weight (kg)	109.6 (17.7)	110.8 (18.4)	0.715		
BMI (kg/m^2^)	38.2 (4.5)	38.0 (4.6)	0.773		
Waist circumference (cm)	107.8 (12.3)	107.2 (13.2)	0.774		
VO_2max_ (mL·kg^-0.75^·min^−1^)	85.0 (14.6)	97.0 (19.1)	< 0.001		
VO_2max_ (mL·kg^−1^·min^− 1^)	26.4 (4.9)	30.0 (6.1)	< 0.001		
VO_2max_ (L·min^−1^)	2.86 (0.57)	3.30 (0.76)	< 0.001		
1-yr					
Weight (kg)	109.4 (19.2)	108.8 (21.3)	0.845		
BMI (kg/m^2^)	38.1 (5.3)	37.2 (5.8)	0.348		
Waist circumference (cm)	106.8 (14.4)	104.9 (15.5)	0.446		
Changes 1-yr					
Weight change (%)	−6.4 (−8.1, −4.7)	−9.7 (−11.7, −7.7)	0.014		
Weight change (kg)	−7.5 (−9.6, −5.5)	−11.4 (− 13.8, −9.1)	0.013	4.0 (0.9, 7.1)	0.012
BMI change (kg/m^2^)	−2.6 (−3.3, − 1.9)	−3.9 (− 4.8, −3.1)	0.013	1.4 (0.4, 2.5)	0.010

Data are presented as mean (SD or CI 95%) or numbers (%). Groups are divided by median percentage increased VO_2max_ median (11.6 percentage) from baseline to 3- month follow-up. Differences between groups analyzed using independent samples t-test or Fisher’s exact test (gender) as appropriate. Adjusted differences between groups in term of changes were analyzed using analysis of covariance (ANCOVA) including age, gender and baseline value of outcome variable as covariates

## Discussion

In accordance with our hypotheses, patients with higher (above median) CRF before treatment achieved significantly greater 3-month and 1-year average weight loss; 2.5 kg and 4.0 kg, respectively, than those with lower CRF. Further, those with larger (above median) initial 3-month improvements in CRF had on average 4.0 kg greater 1-year weight loss than those with smaller CRF changes. In addition, the proportion of patients achieving a weight loss of ≥10% after 3 months was significantly higher in those with higher CRF at baseline, while the proportion of patients achieving a weight loss of ≥10% after 1 year was higher among the patients who achieved larger initial 3-month improvement of CRF.

To the best of our knowledge, this is the first study of patients with severe obesity undergoing an ILI program which assesses the potential predictive effects of pre-treatment CRF and initial changes in CRF on achieved weight loss. A previous cross-sectional study reported that both lower resting metabolic rate and CRF were associated with higher BMI, but the study design precluded any assessment of potential effects of CRF on weight loss [3]. The significant association between baseline CRF and weight loss at the 3-month and 1-year follow-up in the present study may be explained by the potential of greater energy expenditure in patients with higher CRF [25].

The measurement of CRF as VO_2max_ [20], the gold standard for measurement of indirect calorimetry, and the number of patients undergoing a 1-year ILI program, strengthen the results. Since the ILI program also included energy restrictions, the weight loss results might also have been affected by this. We cannot exclude the possibility that the patients with the greatest improvement in CRF may also have followed diet restrictions most rigidly. Unfortunately, dietary compliance was not registered. Further, CRF may influence the ability to perform non-exercise activity and reduce the number of hours of inactivity, or vice versa, thus increasing weight loss [26–28]. On the other hand, higher body weight could also have limited the non-exercise activity and exercise activity and thus limited the potential to increase CRF. However, the present study did not assess non-exercise activity or exercise activity.

This observational cohort study may also have other limitations. First, only treatment-seeking patients with severe obesity were included, thus limiting the generalizability of the results to similar populations. Second, the study population consisted of predominantly white patients, therefore limiting the generalizations to other ethnic groupings. Thirdly, initial baseline values, age and gender differed slightly between groups below or above median. However, these differences were minimized by adjustments for initial baseline values, age and gender. Fourth, it is possible that patients who were more successful in increasing CRF and/or decreasing body weight were more likely to attend follow-up sessions. This may have led to an overestimation of the possible effect of improved CRF. Finally, patients detailed calorie intake and compliance with the prescribed calorie restriction in the ILI program were not assessed.

If verified, our results may be generalized to similar ILI programs in public health care systems. The possible effect of CRF on weight loss and energy expenditure should, however, be examined further. If increasing CRF causes weight loss in randomized clinical trials, exercising to improve CRF should be considered as a natural part of future ILI programs in patients with severe obesity. Importantly, increasing CRF has substantial cardiovascular health benefits, regardless of its effect on weight loss [29].

## Conclusion

Among patients with severe obesity who underwent a 1-year ILI program, higher baseline CRF was associated with significantly larger weight loss after 3 months and 1 year. In addition, those with higher initial 3-month CRF changes had greater weight loss at 1 year.

## Additional files

Additional file 1: Intervention. (PDF 99 kb)

Additional file 2: Supporting information. (PDF 43 kb)

## Data Availability

The datasets used and/or analysed during the current study are available from the corresponding author on reasonable request.

## Abbreviations

BMI: Body mass index
CRF: Cardiorespiratory fitness
ILI: Intensive lifestyle intervention
VO_2maz_: Maximal oxygen consumption
