# Supplementary material for: Associations between cardiorespiratory fitness and weight loss in patients with severe obesity undergoing an intensive lifestyle intervention program: retrospective cohort study
Source: BMC Endocr Disord. 2019 Jul 1;19:69. doi: 10.1186/s12902-019-0394-z (PMC6604389; doi:10.1186/s12902-019-0394-z)
Supplement: Supplementary file 1 — Intervention. (PDF 99 kb) [file 12902_2019_394_MOESM1_ESM.pdf]

Associations between cardiorespiratory fitness and weight loss in patients with severe obesity: retrospective cohort study

Jarle Berge[1,2,3], Øyvind Støren[3], Jens K. Hertel[1], Espen Gjevestad[1, 2, 4], Milada Cvancarova Småstuen[1], Jøran Hjelmesæth [1,5].

1. Morbid Obesity Centre, Vestfold Hospital Trust, Tønsberg, Norway. 2. Clinic Medicine and Rehabilitation, Vestfold Hospital Trust, Tønsberg, Norway 3. Nature, health and environment, University of Southeast, Bø in Telemark, Norway. 4. Norwegian Police University College, Stavern, Norway. 5. Department of Endocrinology, Morbid Obesity and Preventive Medicine, Institute of Clinical Medicine, University of Oslo, Oslo, Norway

Correspondence: Jarle Berge, Vestfold Hospital Trust, Box 2168, 3103 Tønsberg, Norway. Email: Jarle.berge@siv.no

Additional file 1

Intervention

The ILI included both dietary and physical interventions. Patients received a dietary plan with an energy restriction of 1000 kcal d^{-1} of the calculated total energy expenditure, with the specific aim of at least a 5–10 % weight loss. This was otherwise compiled according to Norwegian nutritional guidelines. The first year of the intervention was divided into two stages: The first 12 weeks included the most intensive treatment period with treatment sessions 3 days a week. Each treatment day lasted 6 hours, with patients participating in two supervised training sessions for 60–90 min. This was followed by lectures on nutrition, physical activity and motivation. The first exercise session each day included weight-bearing activities such as walking, Nordic walking, running, ball games, resistance training and various other exercises. The second exercise session consisted of water aerobics, swimming and various other physical water-based activities. The main part of the physical training was aerobic endurance training of moderate (four metabolic equivalents [METs]) to high intensity (8 METs). The resistance training consisted of 10–15 repetitions and 2–3 sets per exercise. Study participants had individual sessions with qualified personnel who used a lifestyle modification intervention in order to invoke behavioral change in the participants. During weeks 13–52, patients received monthly follow up, alternating between group-based and individual sessions every other month. During the group-based sessions patients performed various water-based endurance exercises for the first hour, followed for the next 2 hours by a lifestyle modification intervention including nutritional and physical activity lectures. To calculate each patient's basal metabolic rate (BMR) Schofield's equation was used. Total energy expenditure was calculated by multiplying BMR with an activity factor of 1.4 METs, which is considered to be the increased energy cost of sedentary behavior. The ILI was group based with a group size of 12–14 patients, and the treatment team included a registered nurse, a medical doctor and physical educators with backgrounds in nutrition, pedagogic and adapted physical activity, the latter of which focuses on the individual differences in physical activity that require special attention. The MLI included outpatient treatment 1 day a week for 10 consecutive weeks, for 3 hour each day. The specific treatment goal for each patient was a long- term weight loss of at least 5–10 % through energy restriction and increased physical activity. Participants were encouraged to attend at least 8 of 10 treatment sessions. The treatment staff included a registered nurse, a physiotherapist, a registered dietician and a medical doctor. Each day of treatment included one session of supervised physical exercise such as aerobics, treadmill walking, step machines, indoor cycling and resistance training. The intensity of the physical endurance training was moderate (4 METs) to high (8 METs). The resistance training was performed with 10–15 repetitions and 2–3 sets per exercise. In addition, participants received lectures on nutrition and physical activity, and were motivated by the multidisciplinary team. During the first 10 weeks, patients participated in two water aerobics sessions. Participants were encouraged to increase their PAL between sessions. After the first 10 weeks, participants had to participate in three additional trainings sessions within the following 6 months. Both treatment programs are part of the public healthcare system in Norway and are offered freely to patients.

Time schedule – Intensive Lifestyle Intervention – stage 1. 1-3 months.			
	Monday	Tuesday	Thursday
09:00	Physical activity	Walking, Nordic Walking	Physical activity
09:30			
10:00	Body weight and anthropometric measures		
10:30		Education (physical activity, nutrition and motivation)	Education (physical activity, nutrition and motivation)
11:00	Education (physical activity, nutrition and motivation)	Lunch	
11:30	Lunch		Lunch
12:00		Water activities	
12:30	Education (physical activity, nutrition and motivation)		Education (physical activity, nutrition and motivation)
13:00			
13:30			
14:00	Water activities	Education (physical activity, nutrition and motivation)	Water activities
14:30			
15:00	End	End	End

Time schedule – Intensive Lifestyle Intervention – group sessions – Stage 2. 3-12 months.	
08:45	Body weight and anthropometric measures
09:30	Water activities
11:00	Education (physical activity, nutrition and motivation)
12:00	End

Ref. Gjevestad E, Hjelmesæth J, Sandbu R, Nordstrand N. Effects of intensive lifestyle interventionand gastric bypass on aortic stiffness: A 1-year nonrandomized clinical study. Obesity (Silver Spring). 2015 Jan;23(1):37-45. doi:10.1002/oby.20880. Epub 2014 Aug 30. PubMed PMID: 25174845.
