# Supplementary material for: Associations between cardiorespiratory fitness and weight loss in patients with severe obesity undergoing an intensive lifestyle intervention program: retrospective cohort study
Source: BMC Endocr Disord. 2019 Jul 1;19:69. doi: 10.1186/s12902-019-0394-z (PMC6604389; doi:10.1186/s12902-019-0394-z)
Supplement: Supplementary file 2 — Supporting information. (PDF 43 kb) [file 12902_2019_394_MOESM2_ESM.pdf]

Associations between cardiorespiratory fitness and weight loss in patients with severe obesity: retrospective cohort study

Jarle Berge[1,2,3], Øyvind Støren[3], Jens K. Hertel[1], Espen Gjevestad[1, 2, 4], Milada Cvancarova Småstuen[1], Jøran Hjelmesæth [1,5].

1. Morbid Obesity Centre, Vestfold Hospital Trust, Tønsberg, Norway. 2. Clinic Medicine and Rehabilitation, Vestfold Hospital Trust, Tønsberg, Norway 3. Nature, health and environment, University of Southeast, Bø in Telemark, Norway. 4. Norwegian Police University College, Stavern, Norway. 5. Department of Endocrinology, Morbid Obesity and Preventive Medicine, Institute of Clinical Medicine, University of Oslo, Oslo, Norway

Correspondence: Jarle Berge, Vestfold Hospital Trust, Box 2168, 3103 Tønsberg, Norway. Email: Jarle.berge@siv.no

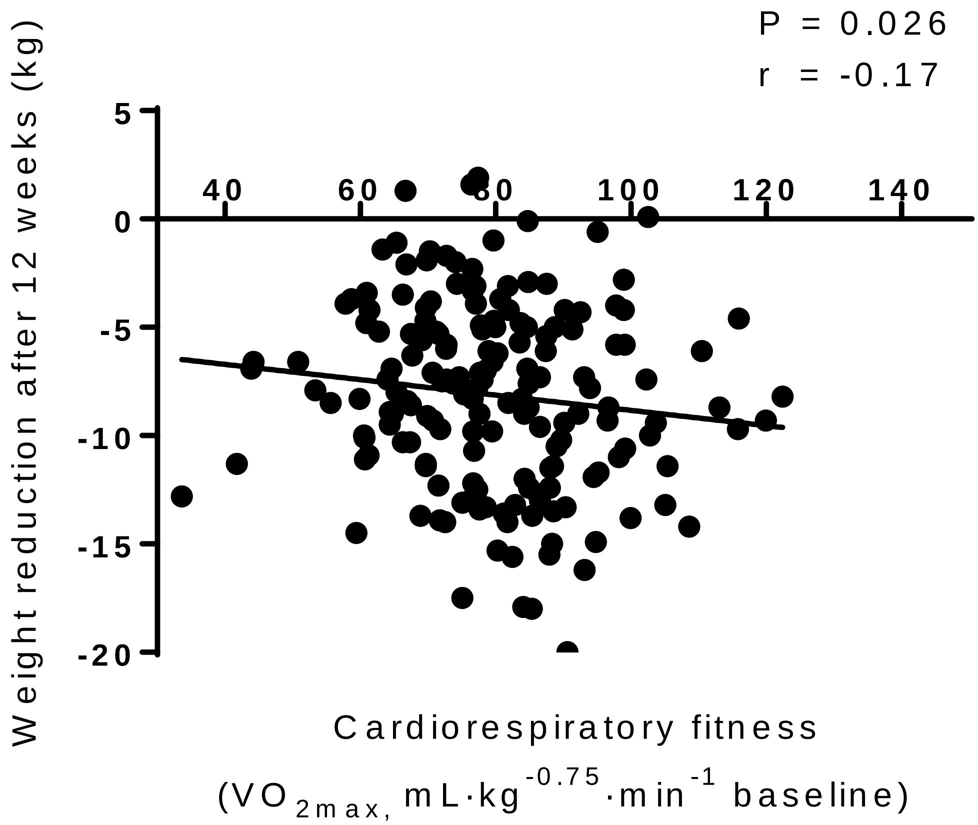
P = 0.026
r = -0.17
Weight reduction after 12 weeks (kg)
5
0
-5
-10
-15
-20
40
60
80
100
120
140
Cardiorespiratory fitness
(VO2max, mL·kg-0.75·min-1 baseline)

Figure S1

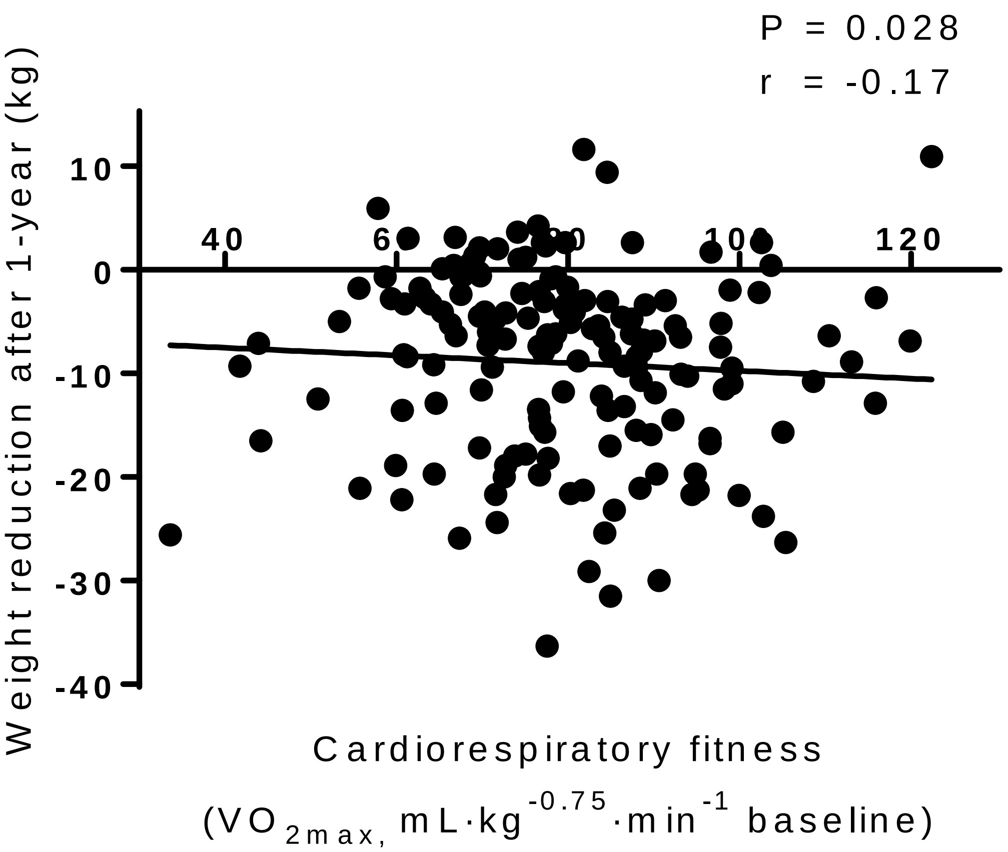
P = 0.028
r = -0.17
Weight reduction after 1-year (kg)
10
0
-10
-20
-30
-40
40
60
80
100
120
Cardiorespiratory fitness
(VO 2max, mL·kg-0.75·min-1 baseline)

Figure S2

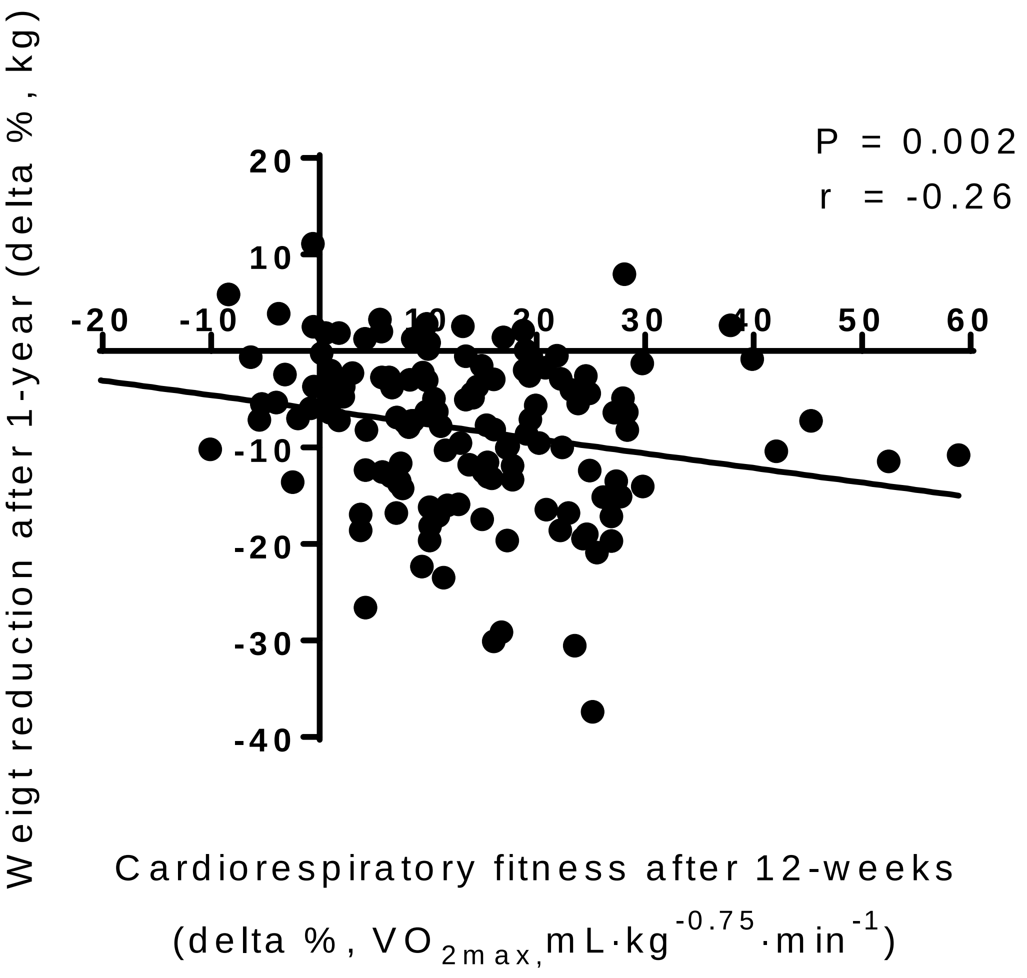
P = 0.002
r = -0.26
20
10
-10
-20
-30
-40
-20
-10
10
20
30
40
50
60
Weigt reduction after 1-year (delta %, kg)
Cardiorespiratory fitness after 12-weeks
(delta %, VO_{2max}, $mL \cdot kg^{-0.75} \cdot min^{-1}$)

Figure S3
